# Health Resorts

**Published:** 1902-11-15

**Authors:** 


### HEALTH RESORTS.

### LEAMINGTON.

Leamington is a town which lends itself well to the purposes of a health resort, its wide streets, its gardens, and its handsome avenues and parades all going to provide an ensemble which distinguishes it at once from any mere residential or business or manufacturing town. It would be an exaggeration to say that the prosperity of Leamington depends upon its waters; perhaps even to assert that its recent growth has been the outcome of their popularity would be going a long way. But the revival which has taken place in the use of the waters, and the considerable expenditure which has been incurred of late in providing proper bathing appliances will certainly add greatly to the prosperity of the town, while, without doubt, its characteristics will greatly add to the attractiveness of the place as a spa. Mildness and equability are claimed as the characteristics of its climate, and it is to be confessed that some people find it relaxing. But it is a pleasant place, with many amusements and plenty going on. A hunting centre also, and one with a certain dash of horsiness all the year round, being the headquarters of the Warwickshire Polo Club. The Leamington waters may be described as a muriated saline containing also a minute quantity of carbonate of iron. Its principal mineral constituent is sodium chloride, which occurs to the extent of 8·5 per mille. Sodium sulphate occurs to the extent of 1·2, and magnesium sulphate to the extent of 0·87 per mille, while there is also a considerable quantity of calcium sulphate. The water when taken in doses of about a pint exerts a slight but certain aperient action. But independently of this action, the salt which it contains seems to be a useful hepatic stimulant, as is generally the case with muriated waters, while by its action on the gastric and intestinal mucous membrane it increases peristalsis and renders the intestinal contents more fluid. These muriated sulphated waters are also believed in some way to improve general metabolism. For external application the Leamington waters seem to act in the same manner as other muriated waters, except that they are far weaker than the more famous springs of this character. Leamington is much resorted to by those who suffer from hepatic disorders, the result of prolonged residence in hot climates; by those in whom dietetic errors, including the over use of alcohol, have induced gastro-hepatic congestion, and by many people suffering from gout and rheumatism in their various forms. A good many people also who suffer from bronchitis and cardiac affections go there in the winter for the sake of the comparative mildness of the climate, which, with the well-kept walks and pleasant parades, gives them opportunity for outdoor exercise which is not always easily obtainable elsewhere. A good deal has been done of late to bring the Pump Room and Bathing Establishment up to date, and it is now possible to obtain there many special forms of treatment, Aix massage, radiant-heat baths, "Nauheim" treatment, medicated baths, wave-baths, etc. The baths are mostly resorted to in spring and summer, but they can be employed at any time. Leamington has of course its winter season also, when hunting men congregate there as well as invalids.

### SIDMOUTH.

Sidmouth is a health resort which may well be borne in mind by those who are seeking protection from the rigours of an English winter, for although the dweller in Sidmouth may at any time obtain the breeziest of fresh air by the simple device of driving up to the moorland on the hills behind the town, he can if he chooses, and he generally does choose, pass the winter well protected from tempestuous winds. Nestling as it does between two steep hills, in a valley which opens to the south upon the sea and is protected from the north winds by the hills which close in the valley in that direction, Sidmouth contains many sheltered spots in which those who desire a protective climate may pass the winter as safely as in any place in England, and more safely than in some of the windy suntraps of the Riviera. It is to be noted, however, that the hills which give to Sidmouth its protection, perhaps from the irregularity of their contour, are apt to divert the winds into unexpected channels, so that those to whom warmth is essential had better make inquiries locally before fixing upon the exact position in which they will take up their winter quarters. The warmth, and perhaps the moisture, of the air is attested by the profusion of evergreen vegetation which is met with in and around the town. The soil of the entire valley in which Sidmouth lies is composed of the marls and sandstones of the Triassic system, but the bottom of the valley is filled in by a deep lay of flinty gravel, and it is on this bed that most of the town stands. The tops of the hills, on the other hand, are formed of the upper greensand, and are useful collecting grounds for the water supply, which is of beautiful softness.

It is the boast of Sidmouth that, although the amount of recorded sunshine for the whole year may be less than in some other places, that for the winter months is greater, so that while the climate is warm in winter, it is not too warm in the summer months. It is said that snow rarely falls in this favoured locality. Even though the hills around may be thoroughly covered with snow, the Sid valley remains green.

In regard to sanitary matters Sidmouth appears to be well looked after. The water supply is derived from the greensand which overlies the marl. The greater part of the catchment area is uncultivated. The water is soft and of good quality, and is supplied on the constant system in considerable abundance. Drainage used to be a difficulty, some portion of the town lying low, and the very porosity of the gravel of these portions of the town complicating the matter considerably. About five years ago, however, the whole drainage of the town was seen to and a modern system installed; the greater part of the sewers being relaid and a collecting tank constructed so as to enable the sewage to be discharged at such a phase of the tide as to be carried away from the town. Since then all seems to have gone well, and for several years in succession the death-rate from zymotic disease has been returned as "nil."

Now as to the cases that go there. It must be remembered that the Sidmouth climate is essentially a sedative and protective one. Kidney disease, gouty patients who require fresh air but cannot keep themselves warm in more bracing places, heart cases, to whom the same remark applies, cases of bronchitis, many of which do admirably, delicate children to whom fresh air is life, and on the other hand elderly people, all these do well. Many cases of early phthisis find the place beneficial, perhaps more particularly those in which the disease is accompanied with catarrh and may be dilatation of the bronchi, and in which there is much expectoration. In the same way also it is a comforting place for more advanced cases in which cure can hardly be looked for. Finally, Sidmouth is a quiet and restful place, and good for those who are worn and sleepless. To all this it must be added that Sidmouth possesses some excellent baths which have been arranged for carrying out the Nauheim treatment for heart disease as well for the administration of Aix massage and other hydrotherapeutic methods used in the treatment of rheumatic affections. It is a gentle, quiet place where, as in everything else so in getting well, one takes one's time.

